# Integrated LTCC Pressure/Flow/Temperature Multisensor for Compressed Air Diagnostics†

**DOI:** 10.3390/s101211156

**Published:** 2010-12-08

**Authors:** Yannick Fournier, Thomas Maeder, Grégoire Boutinard-Rouelle, Aurélie Barras, Nicolas Craquelin, Peter Ryser

**Affiliations:** Laboratoire de Production Microtechnique (LPM), École Polytechnique Fédérale de Lausanne (EPFL), Station 17, CH-1015 Lausanne, Switzerland; E-Mails: thomas.maeder@epfl.ch (T.M.); gboutinardrouelle@gmail.com (G.B.R.); barrasaurelie@gmail.com (A.B.); nicolas.craquelin@a3.epfl.ch (N.C.); peter.ryser@epfl.ch (P.R.)

**Keywords:** LTCC, integrated multisensor, pressure, flow, temperature, SMD mounting

## Abstract

We present a multisensor designed for industrial compressed air diagnostics and combining the measurement of pressure, flow, and temperature, integrated with the corresponding signal conditioning electronics in a single low-temperature co-fired ceramic (LTCC) package. The developed sensor may be soldered onto an integrated electro-fluidic platform by using standard surface mount device (SMD) technology, e.g., as a standard electronic component would be on a printed circuit board, obviating the need for both wires and tubes and thus paving the road towards low-cost integrated electro-fluidic systems. Several performance aspects of this device are presented and discussed, together with electronics design issues.

## 1. Introduction

In the past years, the fields of sensors and microfluidics in LTCC technology have been increasingly studied [2–11], adding new possibilities to this material initially developed for demanding electronics and packaging applications [11–16]. Research has led to the emergence of micro-heaters [5,17], flow [18]/pressure [19]/force [20,21] sensors, micro-reactors, fluidic mixing channels and bioreactors [9,22], However, most of these devices (with some notable exceptions [19,21]) were mainly developed as stand-alone products without integrated signal amplification and conditioning, and hence not suited for industrial applications involving surface mounting technology (SMT).

Many devices of various types are available on the commercial fluidic sensor market offering high precision measurements (≤0.3% of full scale). They usually require physical mounting by tightening and fluidic interconnection with large gas fittings (e.g., G1/2″). The electrical lines transit through connectors such as the DB9 or standard copper wires. These sensors are generally priced well above 100 USD, and are sometimes coupled to regulators (e.g., pressure or flow controllers). Such devices are well suited for high-end applications; however, their performances, dimensions and price are much too high for diagnostics or safety purposes. There is clearly a need for simple, easily mountable, low-cost, low-precision, yet reliable fluidic sensors, in particular to monitor valves and actuators in the pneumatic industry.

Our laboratory had previously developed different kinds of fluidic sensors in LTCC, aimed for the low-cost, mass production industry. For instance, a micro-flow sensor for liquids [23] was integrated into a disposable microreactor driven by external electronics. An SMD pressure sensor with integrated electronics was also demonstrated, followed by a flow sensor demonstrator to determine the most suitable measurement principle (calorimetric or anemometric) [24]. The anemometric principle proved to be sufficient for coarse measurements, *i.e.*, typically required by applications involving diagnostics.

**Figure 1.**
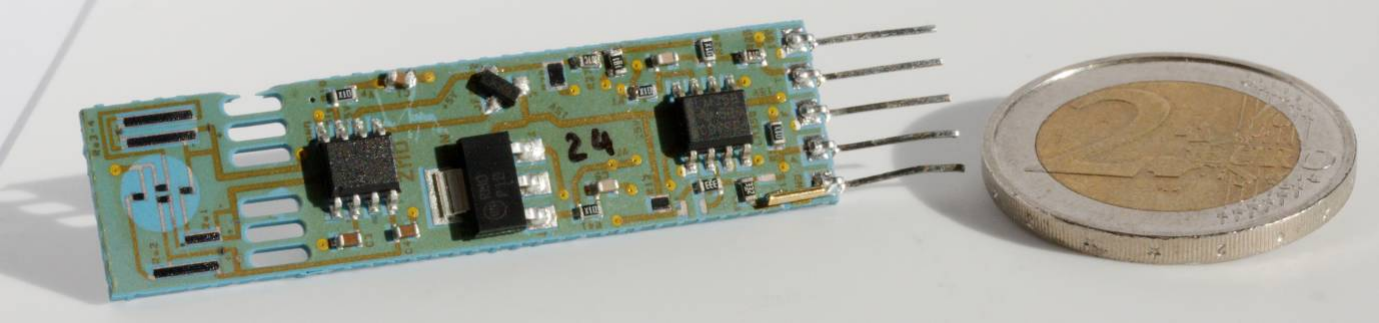
LTCC fluidic pressure-flow-temperature multisensor, with integrated signal conditioning electronics and solderable as an electro-fluidic SMD component on its bottom face (Figure 9). The five pins on the right are for test purposes only.

**Figure 2.**
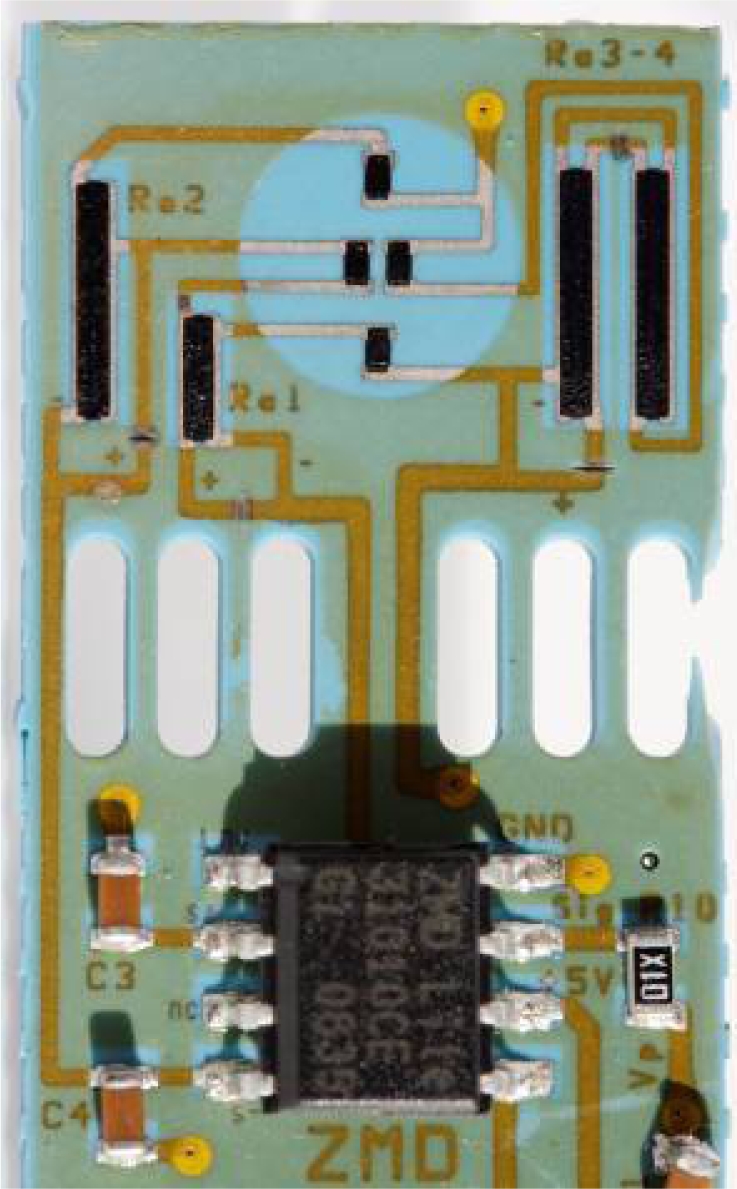
Pressure sensor section with its piezo-resistive bridge and *ZMD* signal conditioning electronics. The outermost right arm was accidentally broken, without impact on performance (see text).

**Figure 3.**
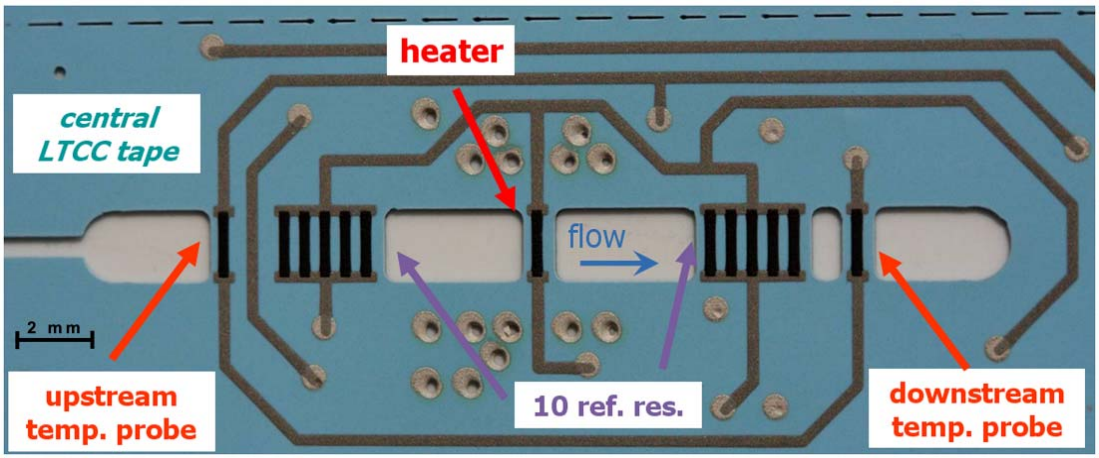
Central LTCC tape (T3) showing the flow + temperature sensor sections. The heater resistor is surrounded by Ag thermal vias; conductor tracks are in Ag:Pd.

In this work we propose, for the first time, a combined SMD sensor in LTCC for measuring gauge compressed air pressure, flow and accessorily temperature, integrating signal conditioning electronics for linearization, adjustment and (for pressure and flow) temperature compensation (cf. Figure 1). The pressure measurement is based on thick-film piezoresistors mounted in Wheatstone bridge on an LTCC membrane (Figure 2); the nominal range is 0…6 bar, with a repeatability of 0.1%, and a precision of 1%. The air flow measurement is based on the anemometric principle, with a heating/sensing thermistor placed in the flow; see Figure 3. The intended range is between 0 and 100 NL/min when using a bypass (only a fraction of the total flow is measured). Finally, two thermistors upstream and downstream give the fluid temperature.

The design of the integrated SMD sensor is described in Section 2, and the manufacturing steps in Section 3. The performances and limitations of each fluidic function are analyzed in Section 4, as well as the LTCC issues encountered.

## 2. Integrated Multisensor Design

### 2.1. Design Guidelines

The integrated sensor was designed with the following guidelines, with the goal of achieving an easily manufacturable and mountable device. Most of the requirements had been validated with our previous prototypes [24].

Pressure sensor principle: piezoresistors in full Wheatstone bridge on a membrane. LTCC must be able to sustain an air pressure of at least 10 bar (nominally 6), in a non-aggressive fluid.

1. Flow sensor principle: anemometric, with 1 heating thermistor suspended on a bridge in the airflow. Aimed range is between 0 and 100 NL/min with a bypass. The reaction time should not exceed 3 s.
2. Temperature sensor: amplification of a resistive bridge comprising thermistors placed toward inlets. The intended range is 0…100 °C.
3. Mounting-induced stresses should not affect the sensor measurements (mainly for the pressure).
4. The device must be compatible with surface mount technology (flip chip). No external wires and no tubes for connections; all connections must be at the bottom, except for test pins.
5. The signal-conditioning electronics must be integrated, yielding only five electrical connections: power, the three sensor output signals, and ground.
6. Laser trimming should be restricted to easy operations: a) coarse trimming of the pressure measurement bridge offset; b) trimming of the differential temperature setpoint of the flow sensing resistor; c) trimming of the temperature signal at room temperature. Cumbersome trimming under pressure or flow, which requires additional fluidic connections to the sensor, should be avoided.
7. Heating of the sensor body by heat flowing into the LTCC from the power transistor and the flow-sensing thermistor must be minimized, which entails providing a good thermal path through the LTCC to well-dimensioned solder pads at the bottom of the device.

#### 

##### Mechanical arrangement

Due to the rather contradictory aspects of the fluidic functions involved, the placement of the sensing elements and the overall shape of the circuit are of capital importance. While the pressure sensor has to avoid heat and mechanical stresses, the thermal flow sensor must be at the same time insulated from external influences, and evacuate parasitic heat efficiently to the outside. Furthermore, the temperature sensor should measure the actual fluid temperature, and not the result of the flow measurement.

**Figure 4.**
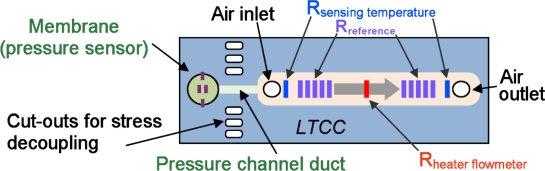
Schematic top view of the integrated sensor, showing the placement of the fluidic functions and the elongated shape of the circuit.

**Figure 5.**
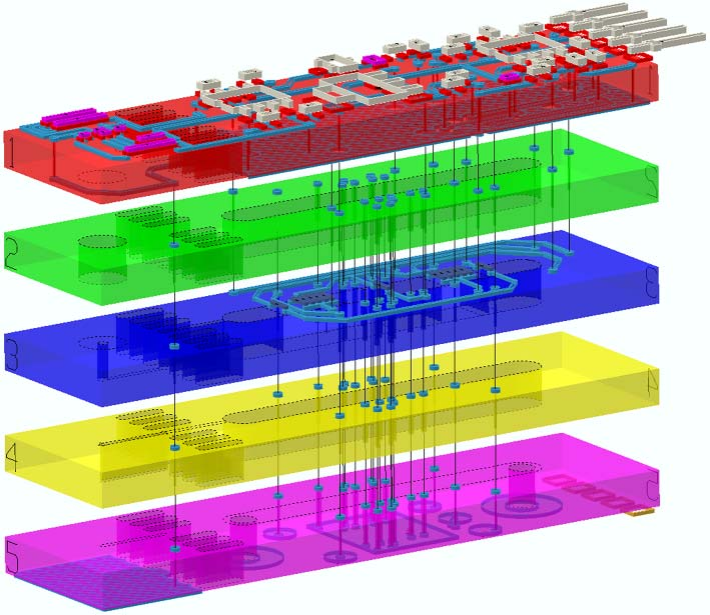
3D, semi-transparent exploded view of the integrated sensor, showing the five LTCC tapes.

These considerations rapidly led to the selection of an elongated shape for the device, as depicted in Figure 4. The fluidic inlet and outlet form the outermost parts of the bottom attachment footprint of the circuit, with the electrical connections lying in between. The pressure sensor is placed free-hanging beyond the outermost attachments to isolate it from mounting stresses. As this free-hanging zone is small in relation to the overall device, mechanical stability remains ensured. Figure 5 displays a semi-transparent 3D view of the sensor.

### 2.2. Flow Sensor Section

**Figure 6.**
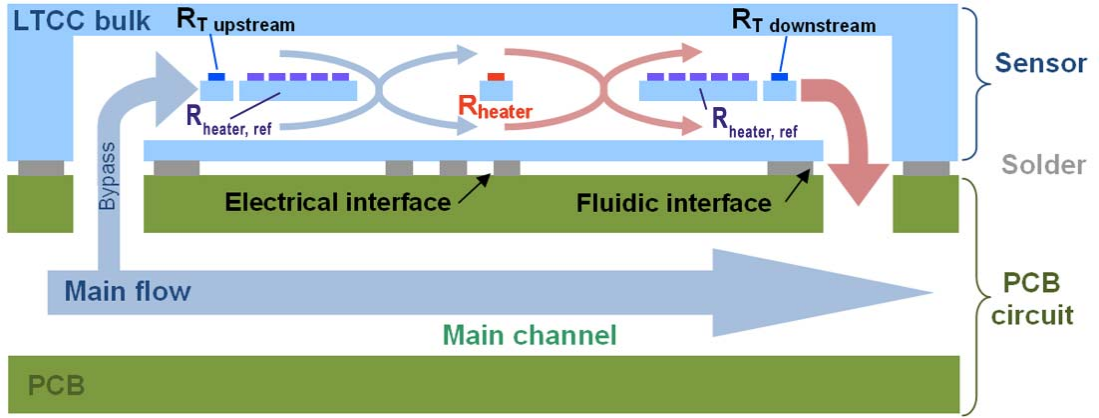
Schematic view of the flow + temperature sensor sections when mounted on a fluidic PCB, depicting the bypass and thermistors.

On the former prototype [24], two thermal mass flow measuring principles were tested: calorimetric (heat diffuses faster than air flows, very sensitive but limited to <5 NL/min), and anemometric (flow goes faster than heat diffuses, less sensitive but compatible with high flows). The latter principle was therefore adopted for the industrial device aimed for in the present work. A bypass arrangement may be used to extend the flow range even further, as depicted in Figure 6.

The flow sensing circuit is a Wheatstone bridge, with one branch consisting in thick-film thermistors in the channel (one heating/sensing resistor R^+^_hi_ + 10 identical ones in parallel forming R^+^_lo_, as reference, cf. Figure 3) and the other branch consisting of fixed setpoint resistors R^−^_hi_ and R^−^_lo_. The use of a single thermistor geometry optimizes the match between the sensing and reference resistors.

**Figure 7.**
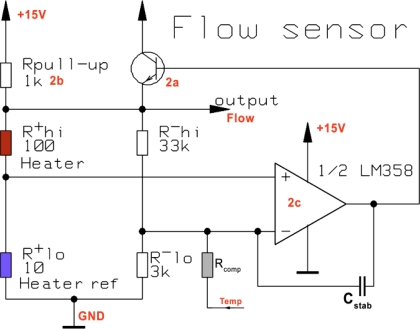
Simplified electrical schematic of the flow sensor section. It is basically a Wheatstone bridge with an amplifier, whose buffered output heats the measuring resistor to regulate its temperature.

We aimed to regulate the central heating resistor R^+^_hi_ ca. 40 K above the reference one R^+^_lo_, estimating this was a good compromise between sensitivity and power consumption. This is done by introducing a controlled nominal imbalance in the bridge, which is actively corrected during operation by the temperature rise. Temperature regulation is carried out by an amplifier (Figure 7) whose output is buffered by a power NPN transistor and fed to the Wheatstone bridge; this bridge supply voltage is also the flow output signal. A minimal bridge supply voltage is ensured by a pull-up resistor in parallel with the transistor.

Several design features were implemented to optimize heat management:

- The heating resistor was thermally decoupled from the LTCC sensor body as much as reasonably possible.
- In order to minimize the effects of the heat leaking from this resistor to the device, its surrounding zone is provided with an array of thermal vias connected to the bottom ground solder pad for heat extraction.
- The NPN power transistor is implanted close to the same thermal vias.
- An Ag ground plane is located under tape T1 to improve heat dissipation from the top SMD components to the bottom solder pads. It however does not extend under the pressure membrane to avoid biasing the piezoresistors.

### 2.3. Pressure Sensor Section

To decouple the pressure membrane from the mechanical and thermal stresses (due to soldering during assembly, heat dissipation, *etc.*), it is advantageous to position it in a cantilever fashion at one end of the circuit, and to place oblong cuts to further mechanically decouple it from the main part of the sensor (Figures 2 and 4).

Measurement of the former demonstrator [24], fitted with a signal-conditioning chip (*ZMD31010*, *ZMDI*, Germany), gave outstanding results: the repeatability of the sensor was better than 0.1%. Its schematics and layout were therefore transposed to the integrated device with only a few changes: lower values of the coarse offset adjustment resistors, and the addition of a JFET transistor (*MMBF 4492*) to get a constant 5 V voltage supply for the piezo-resistive bridge and the *ZMD*, using the latter’s integrated voltage regulation electronics. The whole sensor is supplied at ≥10 V.

### 2.4. Temperature Sensor Section

For the temperature measurement, it was decided to use two thermistors in series, one close to each fluidic connection to measure the average of inflow and outflow and thus be insensitive to flow inversions. This compound thermistor is in series with a fixed one in a half-bridge arrangement, and the resulting signal is amplified.

## 3. Experimental

### 3.1. Sensor Fabrication

Sensors are produced in batches of four per circuit, with three flow channel width variants: 2.0, 2.5 and twice 3.0 mm. The dimensions of a fired sensor are 50 × 12.7 × 1 mm after singulation.

#### 3.1.1. LTCC tapes

As for the previous versions [24], the chosen material is *DuPont (DP) 951 GreenTape™ PX* (254 μm unfired thickness). Thinner tapes (114, 165 µm) are possible choices for the membrane, but to be conservative the 254 μm membrane was selected. Indeed, tests on prototypes with the 254 µm membrane, although implying a weaker bridge signal, proved to be sufficient for diagnostics and performed very well on destructive tests (failure above 24 bar), denoting a safety coefficient >2.4 (relatively to the 10 bar design). In contrast, the 165 μm membranes failed at 12 to 14 bar, yielding a safety coefficient of only 1.2 to 1.4. The maximum deflection of the ceramic membrane upon pressure application was not measured; however, a 30-μm concave sagging due to firing was observed.

The sensor consists of five LTCC tapes (T1–T5), which were sometimes doubled (for a total of five to eight layers) described below:

- T1: top lid (printed on both faces, with the ground plane below)
- T2, T2b: upper channel (vias only)
- T3, T3b: thermistor bridges
- T4, T4b: lower channel (vias only)
- T5, T5b: floor (printed on both faces).

**Figure 8.**
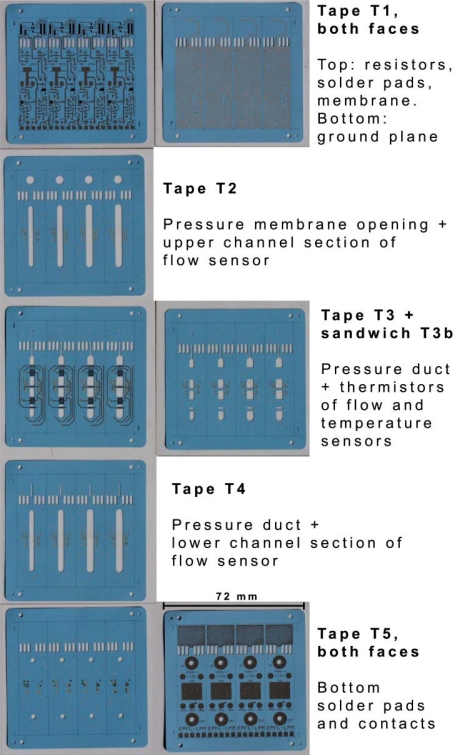
Top view displaying all five LTCC tapes of the integrated sensor (unfired), screen printed and ready for stacking and lamination, with both faces of tapes T1 and T5.

The unfired, printed tapes are all shown on Figure 8. All tapes were 254 μm unless specified otherwise. Some manufacturing variants were produced:

- T3 was tried using 114 or 165 μm thick tape, in order to decrease conduction heat losses.
- T5 was doubled (T5b) to increase lid rigidity (cf. tape issues in next chapter).
- T3 was doubled (T3b) to create a sandwiched version of the thermistors bridges (desirable for media separation, in case gases more aggressive than air must be measured).
- T2 & T4 doubled (T2b, T4b) to increase channel height and increase the distance from bridges to floor & lid.

The retained pastes for cofiring with *DP951* are Ag *DP6141* for vias, Ag:Pd *DP6146* for tracks, pads and resistor terminations, Ag *DP6145* for inner ground plane, *DP2041* (10 kΩ/□) for the piezoresistors, and *DP5092D* (100 Ω/□) for the thermistors. *DPQQ550* was used as a post-firing overglaze, and 96.5Sn-3Ag-0.5Cu as a solder alloy for the components on the sensor.

Ag:Pd was chosen as a termination material in spite of its problematic co-firing behavior [24], as the high thermal conductivity of Au and Ag would induce high thermal losses in the flow-sensing thermistor. Moreover, preliminary tests showed chemical incompatibility between Ag conductors and *DP5092D*.

#### 3.1.2. LTCC processing

After removing the protection tape and pre-conditioning at 120 °C for 30 min, the LTCC tapes were laser cut using a Nd:YAG trimming laser (*LS9000*, *Laser Systems*, Germany; wavelength 1064 nm, 3 W output, 50 μm spot size). The tapes were then screen printed (*900T*, *Aurel*, Italy) in the following successive order, when applicable:

- Via filling (standard vias: holes / catch pads Ø 0.2 / 0.7 mm; thermal vias: Ø 0.4/0.65 mm)
- Conductor printing (linewidths 0.15…0.5 mm);
- Resistor printing (thermistor active length × width = 1.6 × 0.3 mm, piezoresistors 0.4 × 0.4 mm; average measured dry thickness = 30 μm).

Between each print, the tapes were allowed to level at room temperature for 5–10 min and dried for 3 min at 100 °C in an air oven. After printing, the tapes were stacked and uniaxially laminated between protection sheets (*Mylar* or low-density polyethylene–LDPE–foil). No sacrificial volume material was used to prevent deformations and sagging; instead, the process involved a sequence of partial sub-laminations, whose maximal parameters were 100 bar, 46 °C, and 10 min.

After removal of the protective sheets, the stacks were placed on standard alumina substrates and fired in a lamp furnace (*PEO-601*, *ATV*, Germany) with an air flow of 200 L/h. The firing profile was standard, with a long organic burnout to ensure complete combustion (200 min from 250 °C to 450 °C), followed by a 5 K/min sintering ramp and 30 min dwell time at 875 °C. The overglaze (acting as anti-solder mask and protection) was printed and post-fired (510 °C) separately in a belt oven (*2K6-78C52-6AN*, *SierraTherm*, USA).

#### 3.1.3. Mounting of SMD components, singulation and assembly on PCB

**Figure 9.**
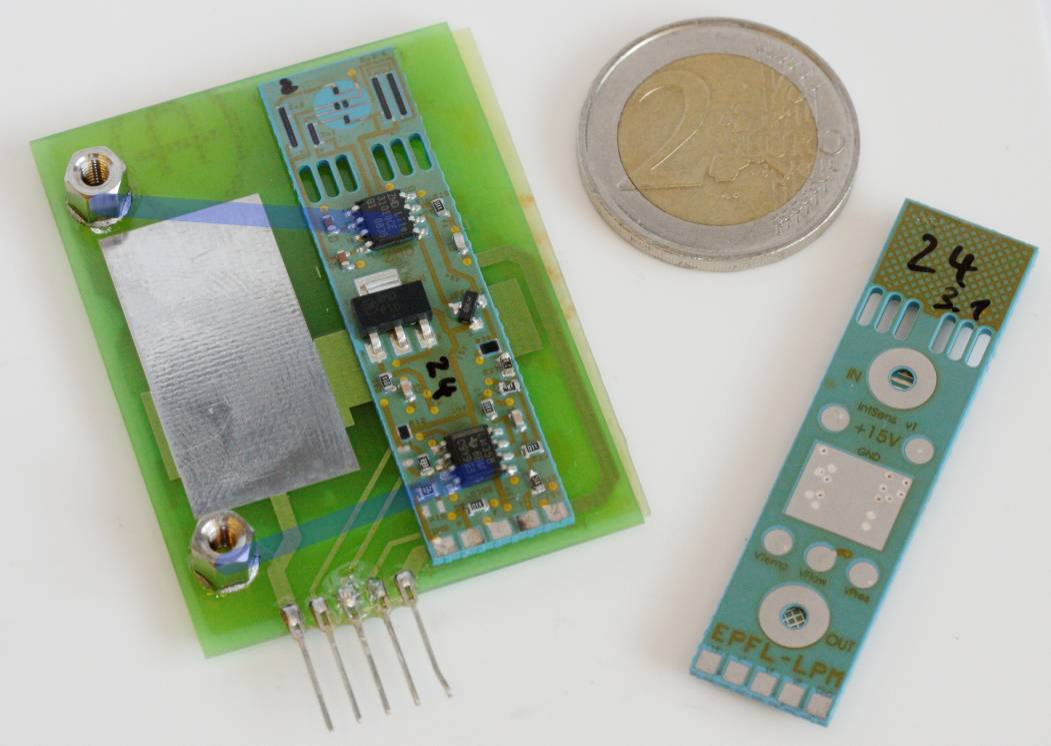
**(Left)** Integrated sensor mounted on fluidic PCB with channels in transparent blue. (**Right)** Bottom face of LTCC sensor showing contact and solder pads, as well as (greenish) overglaze protection.

The SMD components were soldered on top of the substrates using Sn-Ag-Cu solder. This was followed by singulation of the devices, and mounting them onto fluidic test PCBs using a lower-melting Sn-Pb or Sn-Bi solder. These PCBs consisted of three FR4 epoxy plates: base (bottom), channels (middle), and orifices & contacts (top), stacked and glued together. The final result can be seen on Figure 9.

#### 3.1.4. Pressure calibration

First, a coarse adjustment of the raw bridge offset was carried out by digital laser trimming of the adjustment thick-film resistors, to bring the offset within the ∼60 mV/V accepted by the *ZMD* chip. Then, the calibration procedure of the *ZMD* chip was carried out using its *ZMD SSC Evaluation Kit* for programming and a pressure regulator (*DPI520*, *Druck*, Germany, 0…5 bar gauge pressure, precision 0.1 mbar). The pressure signal was set to deliver between 0.5 and 3.5 V for a range of pressures from 0 to 6 bar (gauge). The calibration went as follows:

- Pressure stabilized at 0.0 bar → measurement of first raw bridge value;
- Pressure stabilized at 4.5 bar → measurement of second raw bridge value;
- Computation of the signal conditioning coefficients (gain and offset), and writing into the chip EEPROM.

### 3.2. Measurements

#### 3.2.1. Flow measurements

A flow regulator (*GSC-C4TA-BB26*, *Voegtlin*, Switzerland; 0…20 NL/min, accuracy ±0.3% of full scale) was used as flow reference. No bypass was used in the pneumatic layout, and the PCB was placed downstream of the regulator. Two kinds of measurements were executed:

- At 20 NL/min, the power supply was gradually increased from 0 to 14.8 V to evaluate the behavior of the sensor and determine the nominal temperature rise of the measurement thermistor.
- At constant voltage in normal regulation mode, the flow was gradually varied and the output voltage recorded.

#### 3.2.2. Pressure measurements

The measurements were carried out by connecting the inlet to the regulator and closing the outlet. Ten pressure double ramps (up and down) were applied between 0 and 4 bar in steps of 0.5 bar, with a 5 s wait to allow pressure stabilization.

#### 3.2.3. Temperature measurements

Due to the lack of gas supply with adjustable temperature, it was at the time impossible to test and calibrate the sensor with a flowing gas. Measurements were therefore carried out in an oven (*Heraeus*, Germany) from 30 to 100 °C by steps of 10 K, heating the whole sensor. For each step, the output voltage was first measured directly after powering up the sensor under 12 V, and again after 5 min to assess the influence of sensor self-heating.

## 4. Results and Discussion

This section is divided between structural issues and measurements of each fluidic function.

### 4.1. LTCC Technological Issues

#### 4.1.1. Deformation of suspended bridges

**Figure 10.**
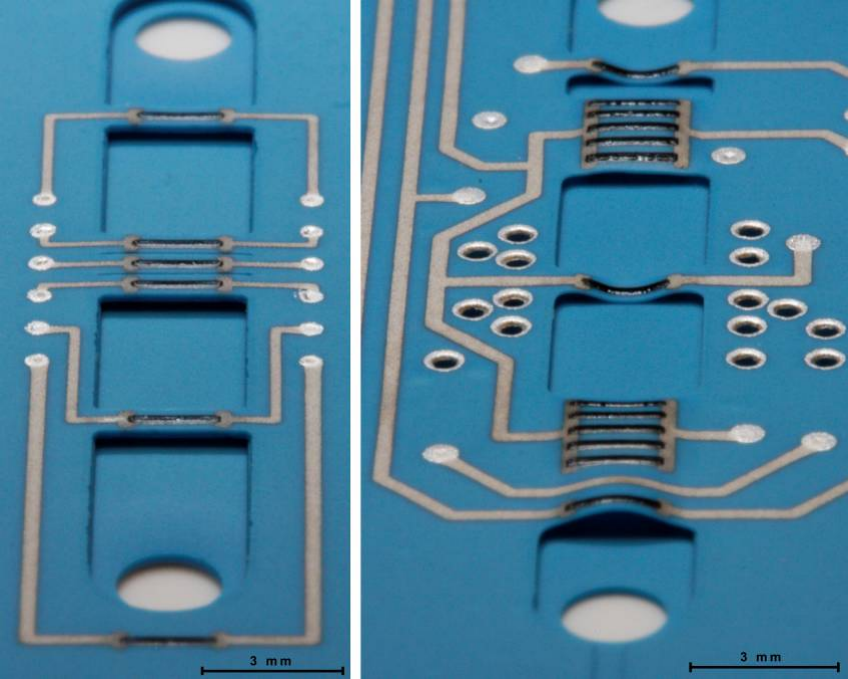
Close-ups on voluntarily incomplete LTCC sensors. **(Left)** Flow sensor prototype [22]: the bridges are intact because Ag:Pd tracks leading to resistors are narrow (0.15 mm), and thus induced deformations are negligible. **(Right)** Integrated sensor: strong deformations of suspended bridges. Note the difference of buckling between resistor blocks, single resistor bridges, and full conductor bridge due to the thick Ag:Pd conductor tracks (0.3 mm).

To verify the integrity of the suspended bridges, the first samples consisted of incomplete LTCC circuits––only tapes T3 to T5 were stacked. The first manufactured bridges exhibited strong buckling, mostly downward, but also upward in some cases, a problem clearly amplified with thin tapes (Figure 10, right). On the contrary, the two parallel resistors blocks yielded less bending, which is thought to be due to the presence of some camber along the channel axis (the wide blocks assume a tile-like shape), which increases their rigidity and makes them less likely to bend. Attempts to induce a “drum-skin effect” [26] (differential shrinkage resulting from applying no lamination pressure to the bridges and high pressure to the surrounding structures) to straighten the bridges failed.

Such issues have been observed before and attributed to differential sintering shrinkage between the LTCC tape and thick-film inks [25,27], with efforts being made to adapt ink shrinkage to that of LTCC [25]. In our case, comparing with our former prototypes [23] which were unaffected (Figure 10 left), we ascribed the bridge deformations to excessively wide (0.3 mm) Ag:Pd conductor lines. Tape T3 was therefore redesigned using the smallest reliable linewidth for all conductors leading to suspended bridges. Also, theses conductors were printed before the via-filling step, in order to avoid line broadening resulting from the deformation of the screen by the filled vias. These changes resulted in straight bridges.

#### 4.1.2. Miscellaneous

**Figure 11.**
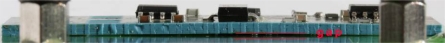
Side view of integrated sensor mounted on PCB depicting convex sensor bending. Note the gap in the middle, which yields a camber of ∼0.4 mm.

- Some complete LTCC sensors exhibited considerable bending in their length, yielding a camber of ∼0.4 mm when laid onto a flat surface (Figure 11). We believe this camber may be due to the large Ag:Pd ground solder pad at the bottom of tape T5, for the same reasons exposed in the previous paragraph.
- The outermost arm of the oblong pressure section cut-outs had a tendency to break up upon manual singulation (Figure 1), due to the singulation stamp-like cuts placed too close to the arm.
- For variants with thinner central tape, a sandwich (by doubling tape T3) was employed to minimize the warping effect. The lamination of this sandwich led to the separation of the tapes, even before the firing: the lamination conditions must still be optimized in order to achieve good interlayer bonding while keeping deformations low.

### 4.2. Flow Sensor Characterization

#### 4.2.1. Characterization *vs.* supply voltage

**Figure 12.**
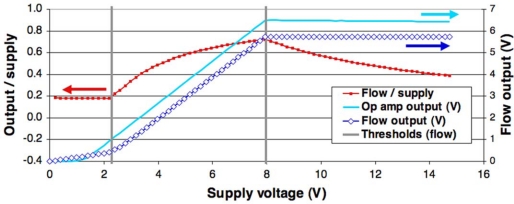
Flow sensor voltage output *vs.* supply voltage for a fixed flow, showing three different behavior ranges.

First, the flow was set at the maximum value the controller could deliver, e.g., 20 NL/min, and the supply voltage gradually increased from 0 to 15 V. The high flow maximizes cooling of the sensing resistor, *i.e.*, allows measurement of the “cold” sensing bridge output. The behavior of a device (#23-2.0) is given in Figure 12. One can clearly see three different supply voltage ranges:

1. 0.0 ... 2.2 V: amplifier not active (*LM358* working at <2 V, but positive saturation & base-emitter voltage drops ≈ 2.1 V);
2. 2.2 ... 8.0 V: amplifier saturated––insufficient voltage to balance bridge;
3. > 8.0 V: normal operation––temperature controlled by amplifier.

The following aspects have to be borne in mind for such sensors:

- The pull-up resistor must have a sufficiently low value to ensure reliable startup of the device, but setting this value too low may cause the resistor, at low flows and high supply voltages, to be heated above its setpoint (*i.e.*, appearance of an unwanted 4th supply voltage range.
- The heating/measuring resistor must be able to reach its setpoint at the highest flow; for a given flow range and sensor design, the bridge supply voltage must be sufficient.
- The *LM358* amplifier is not optimal for this application, due to its ca. 1.5 V saturation voltage drop relative to the positive supply rail and its relatively wide offset specifications; this can be improved by using a rail-to-rail device.
- It is better to use the bridge supply as an output rather than the output of the amplifier, as temperature changes affect the base-emitter voltage of the NPN transistor.

#### 4.2.2. Characterization *vs.* flow

**Figure 13.**
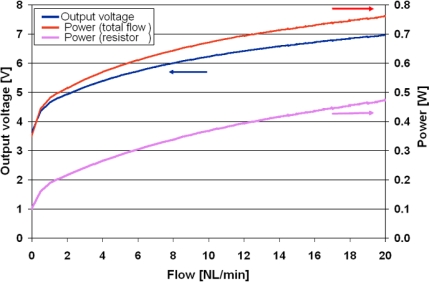
Flow sensor voltage output response and power consumption *vs.* flow for circuit #23-3.1 under 10 V.

The response of device #23-3.1 to flow, under a supply voltage of 10 V, is given in Figure 13. The output *vs.* flow (in blue) is evidently nonlinear, but is well correlated with the dissipated power. The red curve is the total dissipated power of the sensor, while the fuchsia curve is the power dissipated by the heating resistor only. The total dissipation is relatively high, due to the linear bridge voltage regulation.

### 4.3. Pressure Sensor Characterization

**Figure 14.**
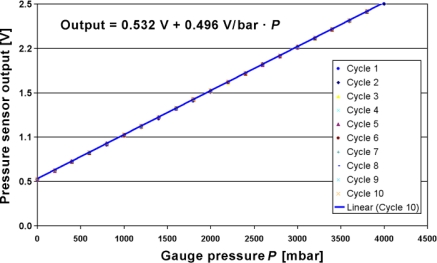
Pressure sensor voltage output *vs.* gauge pressure for 10 cycles between 0 and 4 bar.

**Figure 15.**
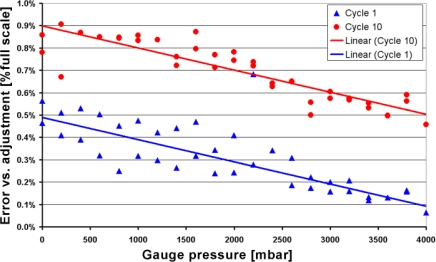
Relative error of pressure sensor voltage output compared to a linear regression *vs.* gauge pressure for cycles 1 and 10.

Figure 14 shows the result of 10 pressure cycles on the signal, and the error *vs.* the original calibration step is plotted on Figure 15 (first and last cycle only). The total error is relatively low (<1%), and slowly drifted upward, an effect attributed to self-heating brought about by the flow sensor part (no flow and poor heatsinking), coupled with the temperature coefficient of the sensor offset (TCO). The selected *ZMD* conditioning chip can compensate TCO, so this source of error can be removed if necessary, although this adds one heating step to the calibration process.

The other main source of error is the slight 0.4% decrease of span between calibration and measurement, which may originate from still imperfect decoupling of mounting stresses. Finally, the residual scatter may lie in the stability range of the pressure regulator.

### 4.4. Temperature Sensor Characterization

**Figure 16.**
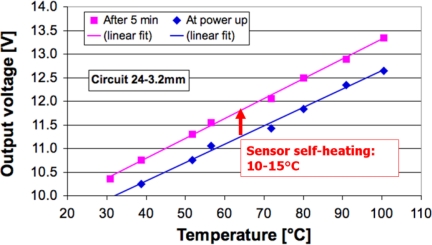
Temperature output voltage *vs.* global sensor temperature, measured at *t* = 0 min since power up (lower line), and at *t* = 5 min (upper line).

The temperature output voltage presents a linear behavior with the global sensor temperature, as expected for this range. The graph of Figure 16 shows this in blue for the measurements immediately after power up, and in fuchsia for the measurements after 5 min. The sensor self-heating can clearly be seen with the vertical shift between the two trend lines, and is estimated to be in the order of 10 to 15 K. Provided the temperature coefficient of resistance (TCR) of the thermistors is well controlled, and the accuracy requirements are relatively lax, one can only trim the sensor offset at room temperature. In case the pressure sensor TCO must be compensated, temperature accuracy can be further improved by trimming sensitivity during the required heating step.

## 5. Conclusions and Outlook

A combined LTCC fluidic multisensor allowing measurement of standard industrial compressed air pressure, flow and temperature with integrated electronics was presented. This innovative device can be mounted with standard surface mount technologies onto an electro-fluidic platform, de facto making it a true electro-fluidic SMD component in itself. It comprises additionally its own integrated SMD electronics, and thanks to standard hybrid assembly techniques, gets rid of external wires and tubings, which was to our knowledge not achieved before. This opens the way for *in situ* diagnostics of industrial systems through the use of low-cost integrated sensors that directly output conditioned signals. The nominal ranges of measurement are 0...6 bar, 0…100 NL/min (tested up to 20 NL/min without bypass), and 0…100 °C, and are well adapted to industrial compressed air. Of course, they can easily be modified to suit other sensing needs.

Even in this first version, the pressure sensor section is already quite satisfactory (an overall precision of 1% is achieved), as well as the temperature sensor part (which uses a “free” amplifier channel, and only requires a simple offset laser trimming step if high precision is not needed). The flow sensor achieves robust and reproducible measurements, but its efficiency is quite low––the total power consumed is typically about three times of that needed to heat the measurement thermistor. The causes and possible solutions for this issue are discussed hereafter:

- **Heating resistor** value too low. The resistance must be designed to fit the combination of temperature differential setpoint and supply voltage, e.g., not to have excessive voltage headroom.
- **Thermal losses between heating resistor and sensor body:**
  ○ Further reduction of conductive and convective losses is still possible by minimizing bridge cross section and area, but fabrication issues must be taken into account.
  ○ A simple way to lower these losses is to decrease the nominal temperature rise of the heater––further tests are needed to determine the resulting compromise between precision and power.
- Inefficient electronics:
  ○ Use of a rail-to-rail amplifier would allow a 1.5 V bridge supply voltage gain (at same power supply voltage), lowering the overall required power.
  ○ More radically, switching to a pulsed-width modulation (PWM) mode of operation would essentially eliminate transistor losses, albeit at the risk of generating interference.

Besides optimization of the flow sensor power requirements, this first version of a fully integrated sensor highlighted many more points that can be improved:

- **Flow and temperature sensor trimming.** The SMD resistors used in this version will be replaced by printed ones, which can be trimmed to calibrate flow and temperature.
- **Overall bending of the sensor.** The sensor design should be changed (partitioning of the ground solder pad) or materials must be tested in order to avoid bending of the whole sensor.
- **Soldering.** The sensor may be soldered with the same Sn-Ag-Cu solder paste as for the top components, which makes it compatible with standard SMD assembly and increases maximum operating temperature. However, this requires better topside solder pad metallizations, as the solder of the top components will melt a second time.
- **Layout.** The layout of the inner ground plane and lines must be improved to suppress bottlenecks, and voltage test points must be added on surface for pressure offset adjustment, and sensor integrity control.
- **Pre-cuts.** The stamp-like pre-cuts allowing singulation by breaking must be changed to avoid destroying the outermost bridges carrying the pressure-sensing structure.
